# Symptomatic splenoma in a child

**DOI:** 10.4103/0256-4947.51780

**Published:** 2009

**Authors:** Parveen Shah, Irfan Robbani, Athar B. Khan, Omar J. Shah

**Affiliations:** a From the Department of Pathology, Sher-i-Kashmir Institute of Medical Sciences, Kashmir, India; b From the Department of Radiology, Sher-i-Kashmir Institute of Medical Sciences, Kashmir, India; c From the Department of Surgical Gastroenterology, Sher-i-Kashmir Institute of Medical Sciences, Kashmir, India

**Figure 1:**
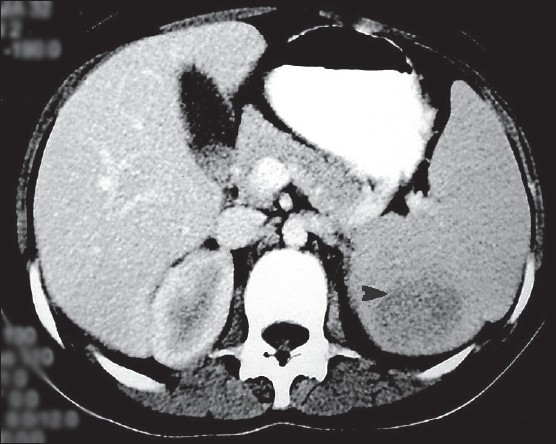
Contrast-enhanced abdominal computed tomography scan demonstrating a low density mass in the lower pole of the spleen (arrow).

**To the Editor:** Splenoma or splenic hamartoma, described in 1861 by Rokitansky, is a rare benign lesion of the spleen, with only 150 cases having been reported in the literature.^1^ They are mostly asymptomatic and are usually discovered incidentally during workup for other unrelated problems or at autopsy. The majority of the reported cases are adult patients; only 20% of cases are in children or adolescents. They have been rarely associated with hematological disorders.^2^ We describe splenoma in a 12-year-old female admitted to our department with symptomatic anemia for the past year in the form of easy fatigability, palpitations and exertional breathlessness. Physical examination revealed pallor and a palpable spleen 5 cm below the costal margin. There was no evidence of jaundice, peripheral lymphadenopathy or hepatomegaly. Contrast-enhanced CT of the abdomen revealed splenomegaly and a 5×6-cm minimal enhancing hypodense mass in the lower pole of the spleen (75-80 Hounsfield units) (Figure 1). CT-guided aspiration of the space-occupying mass lesion proved to be inconclusive. A hemogram showed hemoglobin of 6.8 g/dL (normal, 12-16 g/dL); the white blood cell count was 3.7×10^9^/L (normal, 3.7-12.9×10^9^/L) with a normal differential and platelets of 58×10^9^/L (normal 140-440×10^9^/L). Renal, hepatic and coagulation profiles were within normal limits. The reticulocyte count was 4% and the erythrocyte sedimentation rate was 10 mm/hr. The bone marrow showed hyperactivity suggestive of peripheral destruction of blood cellular elements consistent with hypersplenism. The chest x-ray was normal. The tuberculin test and ANA serology were noncontributory.

**Figure 2:**
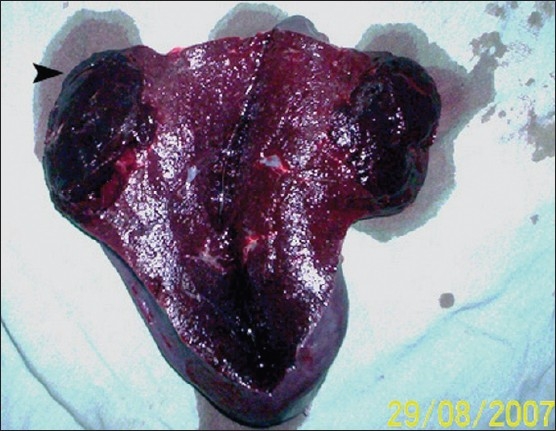
Photograph of the cut section of a resected spleen showing a circumscribed 5×6 cm unencapsulated splenoma (arrow).

**Figure 3:**
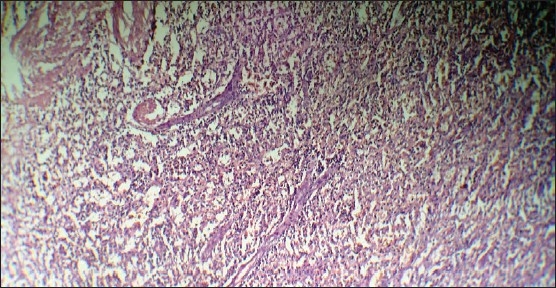
Microscopic image of the splenoma, showing irregularly arranged dilated sinusoids admixed with intervening pulp cord-like elements.

*Pneumococcal* and *Haemophilus influenzae* type b (Hib) vaccines were given. The patient underwent elective splenectomy. Intraoperatively, besides splenomegaly, a bulging spherical mass (5×6 cm) having the same consistency as that of the spleen was detected at the lower pole of spleen. There were no perisplenic lymph nodes and the rest of the laparotomy findings were normal. The postoperative course was uneventful, with complete resolution of both clinical and hematological features. The resected spleen measured 16×10×7 cm and weighed 650 g. On sectioning the spleen, a well-circumscribed mass was identified measuring 5×6 cm at its lower pole (Figure 2). The mass was a bulging, spherical, dark red tissue with the same consistency as the surrounding splenic parenchyma. Histologically, the hamartomatous nodule consisted of splenic sinusoidal tissue with no lymphoid follicles (i.e. red pulp) (Figure 3). In addition, there was variable infiltration by chronic inflammatory cells. Futher, on immunoperoxidase staining, the endothelial cells lining the vascular spaces exhibited reactivity for CD8 and factor VIII related antigens and no reactivity to CD34. A diagnosis of a splenoma was made.

Although the diagnosis is established by a histopathological examination, a preoperative diagnosis using a combination of various modern radiological methods may be possible.^3^–^8^ Sonography is a more sensitive modality than CT in demonstrating the lesion, which usually shows hyperechoic masses with cystic components occasionally.^3^ Sometimes a hypoechoic splenic mass may be found and color Doppler sonography may reveal blood-flow signals within the mass.^4^ Characteristically, CT could reveal splenomegaly and a homogenous or heterogeneous low-density or isodense mass with calcification, or a fatty component.

Dense spreading enhancement on dynamic CT and prolonged enhancement on delayed post-contrast scans are noted in a singular mass,^5^ but low-density masses may be seen in multiple splenomas after contrast medium administration.^3^

Depending on the histological type (fibrous or non-fibrous), MRI findings in splenomas are distinct. In fibrous splenomas, the MRI scan displays isointensity or hyperintensity on T1-weighted images and hypointensity on T2-weighted images,^4^–^7^ whereas non-fibrous splenomas exhibit isointensity on T1-weighted images and hyperintensity on T2-weighted images.^6^,^8^ The tumors can demonstrate diffuse heterogeneous enhancement on the hepatic artery phase or early dynamic contrast-enhanced scans, which became more uniformly enhanced on delayed images.^5^,^8^ Though splenomas have some distinct radiological features, the final and exact diagnosis depends on histopathological examination. Histologically, the characteristic features are irregular sinus-like vascular channels lined by cells that stain positively with CD8 Factor VIII-related antigen and vimentin.^9^ Due to the disordered overgrowth of the red pulp component, splenomas have a variable histological morphology. They should be differentiated from other tumors of the spleen, which include hemangioma, littoral cell angioma, hemangioendothelioma, angiosarcoma and inflammatory pseudotumor. Hemangioma, being the commonest benign tumor of the spleen, is CD8 negative in contrast to the CD8-positive endothelial cells of splenoma. The endothelial cells of littoral cell angioma are CD68 positive and CD8 negative in contrast to splenoma, which is CD68 negative and CD8 positive.^9^

With the use of modern imaging techniques, splenomas may be diagnosed preoperatively, thereby avoiding a splenectomy. While total splenectomy may be necessary if the diagnosis is uncertain or if there is a marked degree of cytopenia or splenomegaly, a partial splenectomy may suffice in some cases.^10^
